# Comparison of acute phase reaction and postoperative stress in pigs undergoing video-assisted thoracoscopic versus thoracotomy pneumonectomy

**DOI:** 10.1186/s13028-016-0256-x

**Published:** 2016-11-09

**Authors:** Hai-Feng Liu, Li Gao, Tao Liu, Yan-Jiang, Hong-Bin Wang

**Affiliations:** 1 Department of Veterinary Surgery, College of Veterinary Medicine, Northeast Agricultural University, No. 59 Mucai Street, Gongbin Road, Xiangfang District, Harbin, 150030 People’s Republic of China; 2 College of Veterinary Medicine, Northeast Agricultural University, Harbin, 150030 People’s Republic of China

**Keywords:** VATS, Pneumonectomy, Acute-phase reaction, Body stress, Postoperative pain

## Abstract

**Background:**

Video-assisted thoracoscopic surgery (VATS) has been used for many thoracic diseases as an alternate approach to thoracotomy. The aim of this study was to compare the surgical outcome of pneumonectomy using VATS with that using thoracotomy pneumonectomy in pigs. Fourteen pigs were equally divided into two groups; one group underwent VATS and the other group underwent transthoracic pneumonectomy. We monitored pre-, intra-, and post-operative physiologic parameters, along with blood cell count, serum C-reactive protein (CRP), serum amyloid A (SAA), interleukin-6 (IL-6) and cortisol. The differences between the two approaches were analyzed.

**Results:**

Mean surgical time in the VATS group (160.6 ± 16.2 min) was significantly longer than that in the thoracotomy group (123.7 ± 13.2 min). In both groups, CRP and IL-6 concentrations were significantly increased at postoperative 4 h, and then gradually decreased to preoperative levels. CRP and IL-6 at postoperative day 1 were significant lower in the VATS group compared with the thoracotomy group. SAA was significantly increased at postoperative days 1 and 3 in both groups compared with preoperative levels. Cortisol was significantly increased immediately after surgery in both groups compared with preoperative levels, and was significantly higher in the thoracotomy group than the VATS group at postoperative 4 h and 1 day.

**Conclusions:**

There was no difference between the two groups in physiologic parameters and blood cell count. However, the results indicate that VATS resulted in a smaller incision, less acute-phase reaction, less stress and less pain compared with thoracotomy pneumonectomy.

## Background

Video-assisted thoracoscopic surgery (VATS) has been used as a diagnostic and therapeutic platform to perform a wide variety of thoracic cavity surgical procedures [1], including surgical lung biopsy, cancer staging, treatment of pneumothorax, pericardiotomy, and pericardial window creation [2]. It is clinically well accepted that VATS is less painful and results in less stress than traditional thoracotomy procedures. Recent reports have also confirmed the safety and benefits of VATS [3, 4].

Surgical procedures are commonly performed on animals as a model for humans. Experimental pneumonectomy has been performed in pigs [5], and the short-term outcome of thoracoscopic lung lobectomy for primary lung tumors has been performed in dogs [6]. Several studies on VATS procedures in animals have evaluated stress parameters and postoperative outcomes [7, 8]; there have also been studies into the outcomes of VATS lobectomy [9].

To thoroughly assess the impact of minimally invasive surgical techniques on acute-phase reactions, stress responses, and postoperative pain, we designed a clinical study comparing such factors in pigs undergoing pneumonectomy via the VATS approach versus thoracotomy to determine the feasibility and safety of both approaches.

## Methods

### Animals

Fourteen healthy Bama miniature pigs with an average bodyweight of 22.57 ± 1.5 kg were used for this study. The pigs were obtained from Bama miniature pigs Farm of College of Life Sciences (Harbin, China). The pigs were housed individually under steady temperature (around 20 °C) and lighting conditions (12 h light/dark cycle), and fed free piglet diet (Shenzhen Jinxinnong Feed, China) and tap water ad libitum. All animals were preoperatively assessed as healthy after physical examination and complete blood count. Animals were randomized and equally divided into two groups: Group I underwent VATS pneumonectomy, and Group II underwent thoracotomy pneumonectomy.

### Surgical preparation

Baseline physiologic parameters were determined just before surgery (t = 0), including routine blood testing, heart rate (HR), rectal temperature, and serum concentrations of C-reactive protein (CRP), serum amyloid A (SAA), interleukin-6 (IL-6), and cortisol.

After a 24 h feeding fast and a 2 h drinking fast, all pigs were administered the same general anesthesia and were monitored and managed similarly. Each pig was premedicated with subcutaneous atropine sulfate (0.04 mg/kg) and intramuscular cefazolin (20 mg/kg). General anesthesia was induced with intramuscular xylazine (1 mg/kg) and ketamine hydrochloride (10 mg/kg), and maintained with 1.5–3% isoflurane in oxygen after intubation. Respiration was maintained via mechanical ventilation with the anesthetic gas machine (respiratory frequency 12 breaths per min, tidal volume 15–20 ml/kg, inspiratory: expiratory ratio = 1:2). Sterile instruments were used for all procedures. Physiological parameters were monitored using an invigilator (MP30; Philips, The Netherlands).

The animal was placed in the supine position. The surgical site (left chest from the clavicle to the last rib and from the sternum to the spine) was shaved, aseptically prepared, and draped for surgery. A heating blanket was placed between the animal and the operating table. All surgical procedures were performed by the same trained veterinary surgeons in the same environment.

Sterile instruments were used for all thoracotomy and VATS procedures. For the VATS procedures, the endoscopes and other equipment underwent high-level disinfection.

### VATS procedure

Two portals were created: the endoscope portal (portal 1) and the surgical portal (portal 2). Portal 1 (1 cm diameter) was created by a trocar inside a metal tube, and was located between the eighth and the ninth rib. Portal 2 (2–3 cm diameter) was created using a scalpel and an endotherm knife, and was located between the fifth and the sixth rib. A 10/11 mm trocar-cannula unit (Optcla Medical Instrument Co. Ltd., Hangzhou, P. R. China) was inserted through the chest wall at portal 1. An endoscope (0°, 10 mm diameter, 330 mm long; Olympus, Hamburg, Germany) attached to a video endoscopic camera and light source (Olympus) was then advanced through this tube into the chest cavity. The margin of portal 2 was treated with an electric coagulation knife for hemostasis.

Lung-grasping forceps were used to lift the lung lobe and expose the hilum of the lung. The pulmonary artery, pulmonary vein, and the bronchus were carefully separated using hemostatic forceps and a homemade tool (peanuts). Electrocautery was used to separate the redundant tissue. The pulmonary artery and pulmonary veins were clipped and transected, and the bronchus was transected after double ligation. The bronchus ligation needed an extra transfixion ligation. All lung lobes in the left chest were excised in the following order: the middle lobe, the upper lobe, and the sub-adjacent lobe. Physiological saline (200 ml at approximately 40 °C) was used to check the bronchus ligation and syringe the thoracic cavity; this liquid was then extracted through a vacuum absorber.

### Thoracotomy procedure

An 8–10 cm incision was created using a scalpel and an electric coagulation knife between the seventh and the eighth rib. The eighth rib was sawed-off to provide a better operating space and view. An endotherm knife was used for blood coagulation. The surgical field was expanded using a wound spreader. The surgical procedure was the same as in the VATS group.

### Monitoring and postoperative care

All animals were reared and monitored in the same environment. The clinical appearance of the pigs, including mental status, movements, appetite, fecal appearance and wound healing, including presence of hemorrhage and inflammation, was evaluated daily for 15 days. Animals were placed in a warm room after anesthesia and transferred to a hog house 6 h later. Freely available water was provided 6 h postoperatively, and pig feed was provided 18 h postoperatively.

A 5 mg fentanyl patch (Duragesic; Janssen Pharmaceutical, China) was applied after surgery, and renewed every 3 for 6 days. Antibiotic prophylaxis consisted of intramuscular cefazolin sodium (50 mg/kg; Harbin Pharmaceutical Group Co., Ltd., General Pharm. Factory, China) twice a day for 2 days.

The following parameters were recorded: HR, rectal temperature, operating time, incision size, postoperative complications, and time of standing up. Hematology examination including white blood cell (WBC), erythrocyte count (EC), lymphocyte (LY), and granulocyte (Gran) count was performed preoperatively and 1, 2, 3, 5, 7, and 14 days after surgery.

Blood samples were collected (preoperatively and 4 h, 1, 3, 5, 7 and 14 days postoperatively,) through the precaval vein, and were centrifuged for 15 min at 1000 g. Serum for biomarkers analysis was stored at −80 °C, and was assayed using commercially available ELISA kits with specific monoclonal antibodies (PharMingen, BD Biosciences, San Diego, CA, USA) according to the manufacturer’s instructions. CRP, SAA, IL-6, and cortisol were measured preoperatively and postoperatively at 4 h, day 1, 3, 5, 7, and 14.

At postoperative day 15, the pigs were euthanized. Pigs were induced with xylazine and ketamine hydrochloride before 300 mg xylocaine was injected intravenously. Necropsy was performed to examine the thoracic cavity condition and postoperative compliance.

### Statistical analysis

Standard statistical methods were used to analyze data. Data were reported as the mean ± SD. Statistical differences within each group were determined by two-way analysis of variance. The paired-sample t test was used to compare the two groups. Significance level was established at *P* < 0.05. Statistical analyses were performed with SPSS software (SPSS Inc., Chicago, IL, USA).

## Results

All animals survived the follow-up period of 14 days. Total pneumonectomy was successfully performed via both VATS and thoracotomy, with complete removal of all left lobes in 14 animals. The operating time for the VATS procedure was significantly longer than for the thoracotomy procedure (VATS 160.6 ± 16.2 min *vs* thoracotomy 123.7 ± 13.2 min, *P* < 0.05).

No animal in either group had intra- or peri-operative complications. There was no evidence of hemorrhage, and no areas of iatrogenic trauma from introduction of surgical instruments. The total length of all skin incisions was 4.5–5.5 cm in the VATS group and 8–10 cm in the thoracotomy group. No other abnormalities were found in either group. All pigs were considered to have returned to their normal activity levels by 24 h after surgery.

**Fig. 1:**
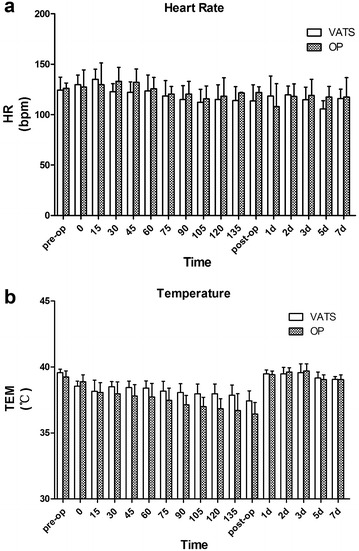
Heart rate (**a**) and temperature (**b**) in 14 pigs that underwent pneumonectomy (*n* = 7 for VATS and *n* = 7 for open thoracotomy). *P < 0.05 compared with preoperative value (baseline). ^*#*^ *P* < 0.05 for VATS vs transthoracic approach at the same time point. *VATS* video-assisted thoracoscopic surgery, *bpm* beats per minute

Rectal temperature was measured before surgery (baseline) and at the designated intra- and post-operative time points (Fig. 1b). Rectal temperature was decreased in all animals intraoperatively and there was no significant difference between the two groups at any time point. Two pigs in the VATS group and four pigs in the thoracotomy group experienced hyperpyrexia on postoperative day 1 and 2, and returned to baseline 5 days after surgery.

**Fig. 2:**
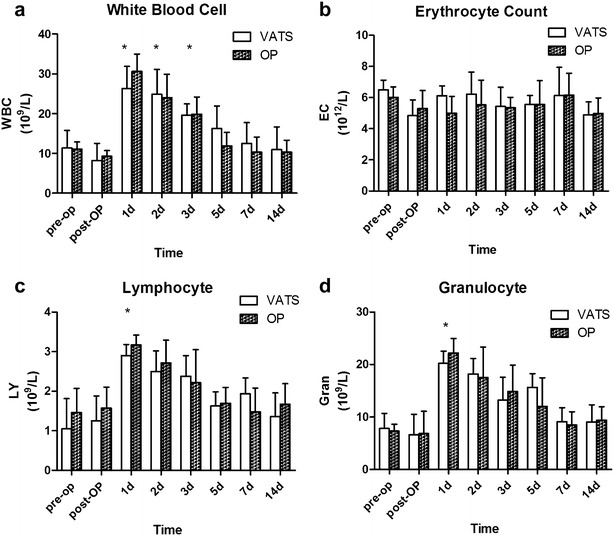
White blood cell count (**a**), erythrocyte count (**b**), lymphocyte count (**c**), and granulocyte count (**d**) in 14 pigs that underwent pneumonectomy (*n* = 7 for VATS and *n* = 7 for open thoracotomy). *P < 0.05 compared with preoperative value (baseline). ^#^ *P* < 0.05 for VATS vs transthoracic approach at the same time point. *VATS* video-assisted thoracoscopic surgery

The WBC count in both groups was significantly increased at postoperative days 1, 2, and 3 compared with preoperatively (all *P* < 0.05), and returned to normal level 2 weeks after surgery (Fig. 2a). There was no significant change in erythrocyte count values after the procedure in either group (Fig. 2b). The LY count and the Gran count were significantly increased 1 day after surgery in both groups (*P* < 0.05) (Fig. 2c, d). The LY count had returned to normal level at postoperative day 5 and the Gran count had returned to normal level at postoperative day 7.

**Fig. 3:**
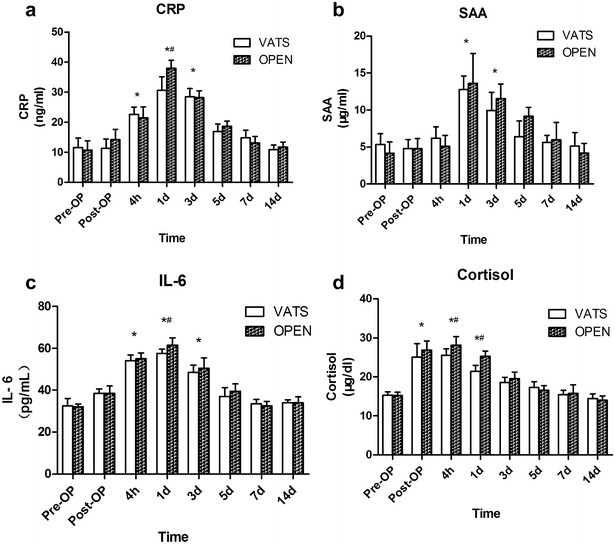
Serum concentrations of CRP (**a**), SAA (**b**), IL-6 (**c**) and cortisol (**d**) in 14 pigs that underwent pneumonectomy (*n* = 7 for VATS and *n* = 7 for open thoracotomy). *P < 0.05 compared with preoperative value (baseline). ^#^ *P* < 0.05 for VATS vs transthoracic approach at the same time point. *VATS* video-assisted thoracoscopic surgery, *CRP* C-reactive protein, *SAA* serum amyloid A

The serum concentrations of CRP in both groups were significantly increased at postoperative 4 h, day 1 and day 3 compared with preoperative levels (*P* < 0.05), and then decreased gradually back to baseline by 7 days postoperatively (Fig. 3a). CRP at postoperative day 1 in the VATS group was significantly lower than that in the thoracotomy group (*P* < 0.05).

The serum concentrations of SAA in both groups were significantly increased at postoperative day 1 and day 3 (*P* < 0.05), and then decreased gradually back to baseline by 7 days postoperatively (Fig. 3b). SAA in the thoracotomy group tended to be higher than that in the VATS group, although this difference was not significant (*P* > 0.05). There was no significant difference in SAA between the two groups at any time point.

The serum concentration of IL-6 in both groups was significantly higher at postoperative 4 h, day 1 and day 3 compared with preoperative levels (*P* < 0.05), and then decreased gradually back to baseline by 5 days postoperatively (Fig. 3c). IL-6 at postoperative day 1 in the VATS group was significantly lower than that in the thoracotomy group (*P* < 0.05), with no difference between groups at any other time point.

The serum concentration of cortisol was increased significantly immediately postoperatively compared with preoperative levels in both groups (*P* < 0.05), and then decreased gradually back to baseline by 5 days postoperatively (Fig. 3d). Cortisol at postoperative 4 h and day 1 was significantly higher than preoperative levels in both groups (*P* < 0.05). The cortisol level was significantly lower in the VATS group than in the thoracotomy group at postoperative 4 h and day 1. No significant difference in cortisol was observed between the two groups at any other time point.

## Discussion

We successfully performed pneumonectomy in the hemithorax via VATS and thoracotomy without major intra- or post-operative complications. Thus, both VATS and the transthoracic approach are viable alternative techniques to total pneumonectomy.

Pneumonectomy is a lung resection technique used in humans and animals to remove all lung lobes when bilobectomy or lobectomy techniques are inadequate to remove the pathology in the hemithorax. Pneumonectomy procedures are performed under some pathological conditions such as lung tumors, congenital lung anomalies, chronic lung collapse, chronic progressive lung inflammation, post-traumatic diffuse parenchymal laceration, and bronchial rupture. Lung lobes can be excised through either thoracotomy or VATS.

Since the first pioneering lobectomy was carried out by VATS [10, 11], the method is now well established as an alternative to thoracotomy for major resections of lung cancer and benign disease [12, 13]. Recent studies have shown that VATS results in a lower concentration of inflammatory cytokines [14], lower risk of developing chest infection, reduced pain, and better lung function than thoracotomy [15, 16].

In the present study, there was a decrease in rectal temperature postoperatively. The central nervous system is restrained in animals undergoing general anesthesia, which results in a decrease in body temperature, HR, respiratory rate and blood pressure. The longer the duration of general anesthesia, the greater the decrease. So the longer surgical time in the VATS group compared with the thoracotomy group led to the lower postoperative body temperature, HR, respiratory rate and blood pressure.

The concentration of CRP and IL-6 in the VATS group was significantly lower than that in the thoracotomy group. The same trend has been found previously in VATS lobectomy [14]. CRP increases rapidly after surgery and indicates the degree of injury [17]. The severe trauma caused by surgery and anesthesia were the reasons for the significant postoperative increases in WBC, CRP and IL-6; however, in pigs, the longer incision in the thoracotomy group seemed to cause more inflammatory reaction than the longer operating time in the VATS group. Although the VATS group had a longer operating time, suturing the incision took more time in the thoracotomy group. Cortisol was significantly lower postoperatively in the VATS group compared with the thoracotomy group. Similarly, cortisol in patients who experienced VATS is reportedly lower than in those undergoing traditional thoracic surgery [18, 19]. It is therefore suggested that thoracotomy causes more postoperative pain and body stress than VATS.

Standard pleural drainage was not used in our study. We did not observe leakage or pneumothorax in any pig. Chest drain use is still controversial, as thoracotomy incisions could damage the intercostal nerves and lead to chronic neuropathy [20]. Satherley et al. [21] reported that the use of an intercostal chest drain after lung biopsy increased the period of hospitalization, and Nakashima et al. [22] reported that postoperative morbidity did not increase after thoracoscopic lung wedge resection without a chest tube. Luckraz et al. [23] reported that an intercostal chest drain was not needed in patients that had received VATS lung resection if no air leakage was noted at surgery. Intraoperative blood loss was not recorded in the current study, as there was very minimal intraoperative bleeding in both groups; however, the blood loss observed in the VATS group was subjectively less than in the thoracotomy group. The reason for this may be the better visual field and accuracy of operation because of the magnified image provided by the endoscope in VATS.

## Conclusions

The use of a minimally invasive surgical technique (VATS) in pigs reduced the acute-phase response, surgical stress and postoperative pain. The VATS approach is the optimal option for thoracic surgery in pigs.

## Abbreviations

CRP: C-reaction protein
EC: erythrocyte count
Gran: neutrophilic granulocyte
HR: heart rate
IL-6: interleukin- 6
LY: lymphocyte
SAA: serum amyloid A
VATS: video-assisted thoracoscopic surgery
WBC: white blood cell
